# Dr. S. H. Smith’s Artificial “Spa”

**Published:** 1859-08

**Authors:** 


*The Spa.*—Under this name, Dr. S. Hanbury Smith has established, at 833 Broadway, near 13th street, fountains of artificial mineral waters, several of the most valuable of the German springs being reproduced as regards chemical composition and temperature. Four springs, models of different classes, have been selected by Dr. Smith, and the waters exactly imitated. They are, *first*, the *Carlsbad* spring, which is hot and alkaline, the sulphate of soda being the largest medicinal ingredient; *second*, the *Manenbad*, which is cold, and resembles closely, in other respects, the Carlsbad; *third*, the *Kissingen*, in which the muriate of soda is the most prominent ingredient, resembling, in this respect, the Saratoga waters; and, *fourth*, the *Pyrmont*, a chalybeate spring.

In Europe, artificial mineral waters, first established by the eminent chemist, Berzelius, are much in vogue; and experience appears to show that the therapeutical effects fairly attributable to the natural springs, may be obtained by their counterparts as prepared artificially. Exclusive of the adjunctive influences of change of scene, exaggerated faith, etc., it is certainly reasonable to conclude that the same remedies, similarly combined by the art of the chemist, will have the same remedial effects, as when found in natural combinations; and that the latter may be in all respects imitated, is unquestionable. Dr. Smith has given to this subject, of late years, much attention, and has brought the manufacture of the different waters to great perfection. In bringing them to the notice of the public, he resorts to none of the tricks of quackery. He claims for his artificial waters no more than belongs to them as faithfully representing the original springs which he professes to reproduce.

The enterprise is a novel one in this country, and merits the attention of physicians. Dr. Smith will cheerfully give information respecting the waters to those who are sufficiently interested to call and examine his establishment, or he will send a brief, printed account of them to those who reside at a distance. All the waters being carbonized, they are drawn from fountains; and they are also put up in bottles.

